# Identification of function-regulating antibodies targeting the receptor protein tyrosine phosphatase sigma ectodomain

**DOI:** 10.1371/journal.pone.0178489

**Published:** 2017-05-30

**Authors:** Chia-Lun Wu, Serge Hardy, Isabelle Aubry, Melissa Landry, Allison Haggarty, Horacio Uri Saragovi, Michel L. Tremblay

**Affiliations:** 1 Rosalind and Morris Goodman Cancer Research Centre, Montréal, Canada; 2 Department of Experimental Medicine, McGill University, Montréal, Canada; 3 Departments of Oncology, Pharmacology & Therapeutics, McGill University, Montréal, Canada; Osaka University, JAPAN

## Abstract

Receptor tyrosine phosphatase sigma (RPTPσ) plays an important role in the regulation of axonal outgrowth and neural regeneration. Recent studies have identified two RPTPσ ligands, chondroitin sulfate proteoglycans (CSPGs) and heparan sulfate proteoglycans (HSPG), which can modulate RPTPσ activity by affecting its dimerization status. Here, we developed a split luciferase assay to monitor RPTPσ dimerization in living cells. Using this system, we demonstrate that heparin, an analog of heparan sulfate, induced the dimerization of RPTPσ, whereas chondroitin sulfate increased RPTPσ activity by inhibiting RPTPσ dimerization. Also, we generated several novel RPTPσ IgG monoclonal antibodies, to identify one that modulates its activity by inducing/stabilizing dimerization in living cells. Lastly, we demonstrate that this antibody promotes neurite outgrowth in SH-SY5Y cells. In summary, we demonstrated that the split luciferase RPTPσ activity assay is a novel high-throughput approach for discovering novel RPTPσ modulators that can promote axonal outgrowth and neural regeneration.

## Introduction

Deregulation of protein tyrosine phosphorylation impacts a broad spectrum of human diseases including obesity, vascular diseases, cancers and neural degeneration [1, 2]. Receptor protein tyrosine phosphatase sigma (RPTPσ) is highly expressed throughout neural development and remains important for neural plasticity even in the adult brain [3–7]. However, the mechanisms underlying the transduction of signals mediated by RPTPσ remain unclear. Recently, extracellular matrix proteins CSPGs (chondroitin sulfate proteoglycans) and HSPGs (heparan sulfate proteoglycans) were identified as ligands of RPTPσ, suggesting a potential mechanism of RPTPσ regulation in nerve injury models [4, 6, 7]. These studies have demonstrated that binding of CSPG to RPTPσ receptors expressed on the axon surface induces axonal retraction [4, 6]. In addition to CSPGs and HSPGs, a number of RPTPσ-interacting proteins have been identified. Several synaptic molecules including TrkC, Netrin-G3, and Slitrk1-3 have been found to interact with the extracellular portion of RPTPσ and are thought to play a role in the regulation of RPTPσ function [8–10].

In contrast to receptor tyrosine kinases, receptor tyrosine phosphatases (RPTPs) are inactive as homodimers. An initial model proposed that a motif of the RPTPs located between the transmembrane segment and the first catalytic domain (D1), named the “wedge domain”, mediates dimer formation and causes RPTP inactivation [11–15]. In this model, the wedge domain of one of the two proteins is proposed to occlude the catalytic site of the D1 domain of the other PTP protein. Several studies support the idea that RPTPσ predominately exists as dimers in vivo [16, 17] suggesting that its ligand(s) regulates receptors dimerization status and thus receptor activity by inducing a radical conformational change of this receptor.

Here, we developed a split firefly luciferase-based sensor system to monitor in real time RPTPσ dimerization/activity in response to various ligands. Using this system, we have validated new anti-RPTPσ antibodies that are capable of modulating axonal outgrowth, and which could be developed as potential therapeutics for the treatment of nerve injury and other neurodegenerative diseases.

## Materials and methods

### Cell culture, antibodies and reagents

293T/17, COS7, SH-SY5Y cells were routinely maintained in DMEM containing 10% Fetal Bovine Serum and 1% penicillin/streptomycin. The following antibodies were used: Anti-PTPRS clone 1H6 (Abnova), Anti-RPTPσ clone 17G7.2 (MediMabs), Anti-tubulin (Sigma). The following reagents were used in this study: Chondroitin Sulfate (Sigma), Heparin (Sigma), Aggrecan (Sigma) and Chondroitin Sulfate Proteoglycan (CSPG) (Millipore), D-luciferin (ThermoFisher Scientific).

### Plasmids and constructs

The murine (NM_011218) and human (BC143287) RPTPσ full-length cDNAs were sub-cloned using PCR-based strategies. The PCR products were digested by EcoRI and SalI restriction enzymes and inserted into pRK5-Nluc, pRK5-Cluc, pRK5-FN and PRK5-CF vectors (gift from David Piwnica-Worms) [18]. The extracellular domain (AA 1–1370) and the DI-DII domain (AA 1370–1904) of RPTPσ without de wedge domain were made by a similar PCR cloning strategy. In addition, since the RPTPσ antibodies used in this study does not recognize the DIDII region of RPTPσ, we included an HA tag on the DI-DII RPTPs N/C constructs to assess their expressions. All plasmid constructs were verified by sequencing.

### Luciferase assay in living cells

293T/17 cells were transfected with Lipofectamine 2000 (Invitrogen) according to the manufacturer’s instructions. 5000 cells were plated in a 96 well plate for 24 hrs followed by transient transfection (0.1 μg/well) of the various constructs. Following transfection, 1 mM D-luciferin in phenol red free medium was added for 30 min at 37°C. Quantification of the luminescence was carried out using a luminometer (FLUOstar Omega) analysed in real-time for a total of 15 min with data collected at 3 min interval.

### Native PAGE

Two micrograms of plasmid DNA was transfected by lipofectamine 2000 in 293T/17 cells. 48 hours post-transfection, cells were treated with purified CSPG then lysed with NP-40 lysis buffer (1% NP-40, 20 mM HEPES, pH 7.4, 150 mM NaCl, 5mM NaF, 1 mM NaPO_4_, 10% Glycerol) containing 1X protease inhibitor (Roche) and 1 mM sodium orthovanadate. Cleared lysates were collected after centrifugation at 12000 rpm for 10 minutes. Protein concentration was determined by BCA (bicinchoninic acid) assay and the lysate supernatants were divided into two portions. One portion was added to 2X SDS-sample buffer (100 mM Tris-Cl (pH 6.8), 4% (w/v) SDS (sodium dodecyl sulfate), 0.2% (w/v) bromophenol blue, 20% (v/v) glycerol, 200 mM β-mecaptoethnol) and the other portion was added to coomassie blue sample buffer (100 mM Tris-Cl, pH6.8, 20% glycerol, 5% Coomassie Briliant Blue g-250). The protein sample with Coomassie blue sample buffer was subjected to native or SDS PAGE (Polyacrylamide gel electrophoresis) followed by standard western immunoblotting procedures as described below.

### Western blot analysis and immunoprecipitation

Proteins were separated by SDS-PAGE and transferred onto polyvinylidende difluoride (PVDF) membrane (Immobilon-P Millipore). The membrane was blocked with 5% BSA in TBST at room temperature then was incubated with primary antibody. For the immunoprecipitation experiment, 500 μg of cell lysates were incubated with the indicated RPTPσ antibodies conjugated to agarose beads for 4 hr. Immunoprecipitated complex were washed with NP-40 lysis buffer for 3 times followed by western blot analysis.

### Membrane protein purification

Six micrograms of RPTPσ expression constructs were transfected into 297T/17 cells in a 10 cm dish using lipofectamine 2000 according to the manufacturer protocol. Ten dishes of transfected cells were rinsed with cold PBS then scraped in 1 ml PBS and collected by centrifugation. Cells were resuspended in membrane wash buffer (0.32 M sucrose, 10 mM Tris-HCl, pH7.5, 5 mM MgCl_2_, 50 mM NaCl) and homogenized using a glass Dounce homogenizer using 25 strokes. Different cellular fractions were separated by ultracentrifugation at 20,000 rpm for 10 min at 4°C. Membrane protein fractions were pelleted and washed 3 more times with the same wash buffer and the concentration measured by BCA assay.

### *In vitro* phosphatase assay

50 μg of the membrane protein fraction overexpressing GFP or RPTPσ N/C was incubated with different amount of Chondroitin sulfate or Heparin at 37°C for 30 minutes followed by the addition of 1 mM p-nitrophenyl phosphate for 30 min. The assay was conducted at 25°C in 96 well plates and the absorbance of p-nitrophenol was monitored at 405 nm every minute using a Varioskan plate reader (Thermo Electron).

### Generation of antibodies against human RPTPσ

Specific sequences in the extracellular domain of human RPTPσ were selected to generate peptides. The KLH-linked RPTPσ peptides were injected into 2 months old RPTPσ KO mice in order to induce antibody-dependent immune response. Four weeks after post injection, mice’s blood was withdrawn for ELISA analysis. Mice positive to ELISA further injected KLH-linked RPTPσ peptides for the second boost of immune response. Mice reached to 3 months old then were sacrificed to take out blood and spleen for hybridoma generation. Mice were euthanized using isofluorane and carbon dioxide followed by cervical dislocation. All animal procedures were approved by the McGill Animal Care Committee and were conducted according to the Canadian Council of Animal Care ethical guidelines for animal experiments.

### ELISA

Epitope peptides from human RPTPσ extracellular region were coated onto 96 well plates overnight. The coated plates were washed with PBS 3 times to remove the excess peptides and 3% BSA solution in PBS was added to the peptide coated plate to block non-specific binding. Supernatant from individual hybridoma medium was added and incubated for 1 hr. Followed by 3 washes, a secondary antibody (1:10000, anti-mouse IgG-HRP) was added, incubated for 1 hr. After 3 washes with PBS, ABTS (2,2'-Azinobis [3-ethylbenzothiazoline-6-sulfonic acid]-diammonium salt) was added and the absorbance read at 410 nm using a Varioskan plate reader (Thermo Electron).

### Flow cytometry

Six micrograms pRK5-hRPTPσ was transfected in COS7 cells using lipofectamine 2000. 24 hrs following transfection, cells were detached using 2.5 mM EDTA treatment and blocked by “Fc blocker” (BD) for 30 min on ice. Primary RPTPσ antibody (1H6) (1:100) or hybridomas supernatant was added to label the epitope on the cell surface for one hour on ice. Cells were then washed with cold PBS containing 2% FBS and labeled with anti-mouse FITC (1:100) for one hour on ice. Labeled cells were subjected to FACS analysis. The result was analysed using Flowjo software (Flowjo).

### Immunofluorescence

SH-SY5Y human glioma cells were treated with 3% FBS, 10 μM Retinoic acid for 96hr in order to induce cell differentiation. After 96 hr, cells were washed with PBS and fixed by 4% paraformaldehyde for 5 min on ice. Cells were incubated with 5% BSA for one hour at RT in order to prevent non-specific binding. Primary antibody (IgG, anti-hRPTPσ 1.3H12, anti-hRPTPσ 4.5H5 at 10 μg/ml each) was applied to cells and incubated for 2 hr at RT. Following PBS washes, cells were incubated with the secondary antibody (1:2000, anti-mouse FITC) for 2 hr at RT. Finally, cells were washed 3 times with PBS and stained with actin dye (Phalloidin) and nucleus dye (DAPI) for 30 min. Samples were mounted and subjected to immunofluorescence analysis.

### Neurite growth assay

SH-SY5Y cells were infected with lentivirus carrying GFP. The infection procedures were described in a previous study [19]. GFP positive cells were treated with differentiation medium, DMEM 3% FBS with 10μM Retinoic acid, for 96hr in presence or absence of 10 ug/ml of the indicated antibodies. Cells were subjected to the image analysis using the NeuronJ program. The length of neurite was measured from the cell body to the tip of the neurite.

## Results

### The RPTPσ split luciferase system

**Fig 1:**
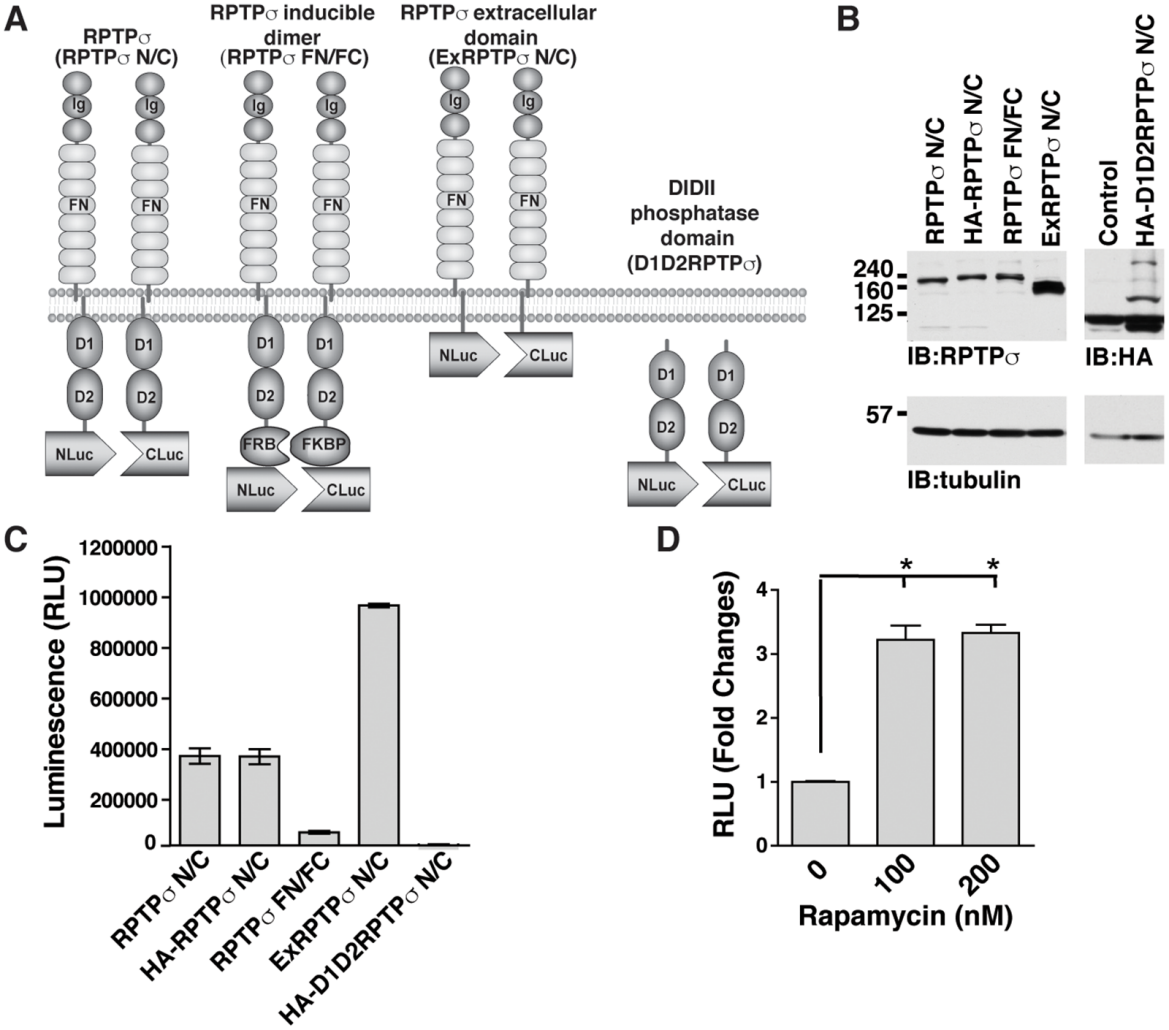
Description and characterization of RPTPσ constructs for the split luciferase assay in live cells. (A) Schematic of RPTPσ constructs used in this study. Different RPTPσ domains were fused with either Nluc or Cluc fragment independently as indicated. FN: Fibronectin domain; Ig: Immunoglobulin-like domain. (B) Western blot analysis following transfection in 293T/17 cells with various RPTPσ constructs. IB: Immunoblot. (C) 293T/17 cells were transfected with various RPTPσ N/C constructs as indicated and 48 hrs post-transfection, relative luminescence unit (RLU) was determined using a luminometer following the addition of 1mM of D-luciferin. (D) 293T/17 cells were co-transfected with RPTPσ FN/FC and 48 hrs post-transfection, rapamycin was added for 4 hr in order to induce FRB and FKBP association followed by luminescence measurement. Values represent fold changes vs control (DMSO) and the mean of three independent experiments performed in triplicates is given ± SD. * p < 0.05.

The split luciferase system is particularly well suited for evaluating real-time receptor/ligand interactions in a cellular context [18, 20–22]. Thus, we first developed a novel assay using this luciferase fragment complementation assay to monitor the dimerization status of RPTPσ in live cells in response to various ligands. We generated several different constructs to assess the contribution of different protein segments of RPTPσ. Amino and carboxyl terminal ends of the reporter luciferase gene were independently fused to the carboxyl-terminal end of full-length, truncated and/or tagged forms of RPTPσ as described in Fig 1A. Following validation of their expression in Hek293T (Fig 1B), the basal luciferase activity of these constructs indicated that both full length and the extracellular domain of RPTPσ displayed higher levels of luciferase activity, indicating that the extracellular domain of RPTPσ is critical for dimer formation (Fig 1C). We also observed a correlation between the expression levels of the extracellular domain of RPTPσ (exRPTPσ) and its luminescence signal when compared to full length RPTPσ. As the extracellular and transmembrane domains are critical for dimer formation of many RPTPs [11–15], we also clearly show here that removal of these regions (DIDII RPTPσ) affects dimerization (Fig 1C), which also confirms the validity of our approach. Importantly, RPTPσ dimerization is not due to transient overexpression artifact since the DIDII domain of RPTPσ had low luminescence activity. Thus, this result also indicates that RPTPσ dimers formation are driven by the presence of pre-existing extracellular ligands including the previously reported RPTPσ ligands, HSPGs which is known to be expressed on Hek293T cell surface [23].

In order to confirm that RPTPσ dimerization can be induced in this assay, we used the FRB-rapamycin-FKBP dimerization system [18] which induces the dimerization of RPTPσ in response to rapamycin. Thus, FRB-Nluc and the FKBP-Cluc domains were fused independently with the full length of RPTPσ (Fig 1A and 1B). Although the presence of the FRB and FKBP domains decreased basal luminescence activity, it was still higher then the DIDII domain of RPTPσ (p = 0.0312) (Fig 1C). Interestingly, after rapamycin treatment, we detected a 3-fold increase in bioluminescence signal in rapamycin treated cells compared to control cells (Fig 1D), confirming the functionality of RPTPσ dimerization split luciferase assay in live cells.

### Modifying RPTPσ dimerization status by its ligands

**Fig 2:**
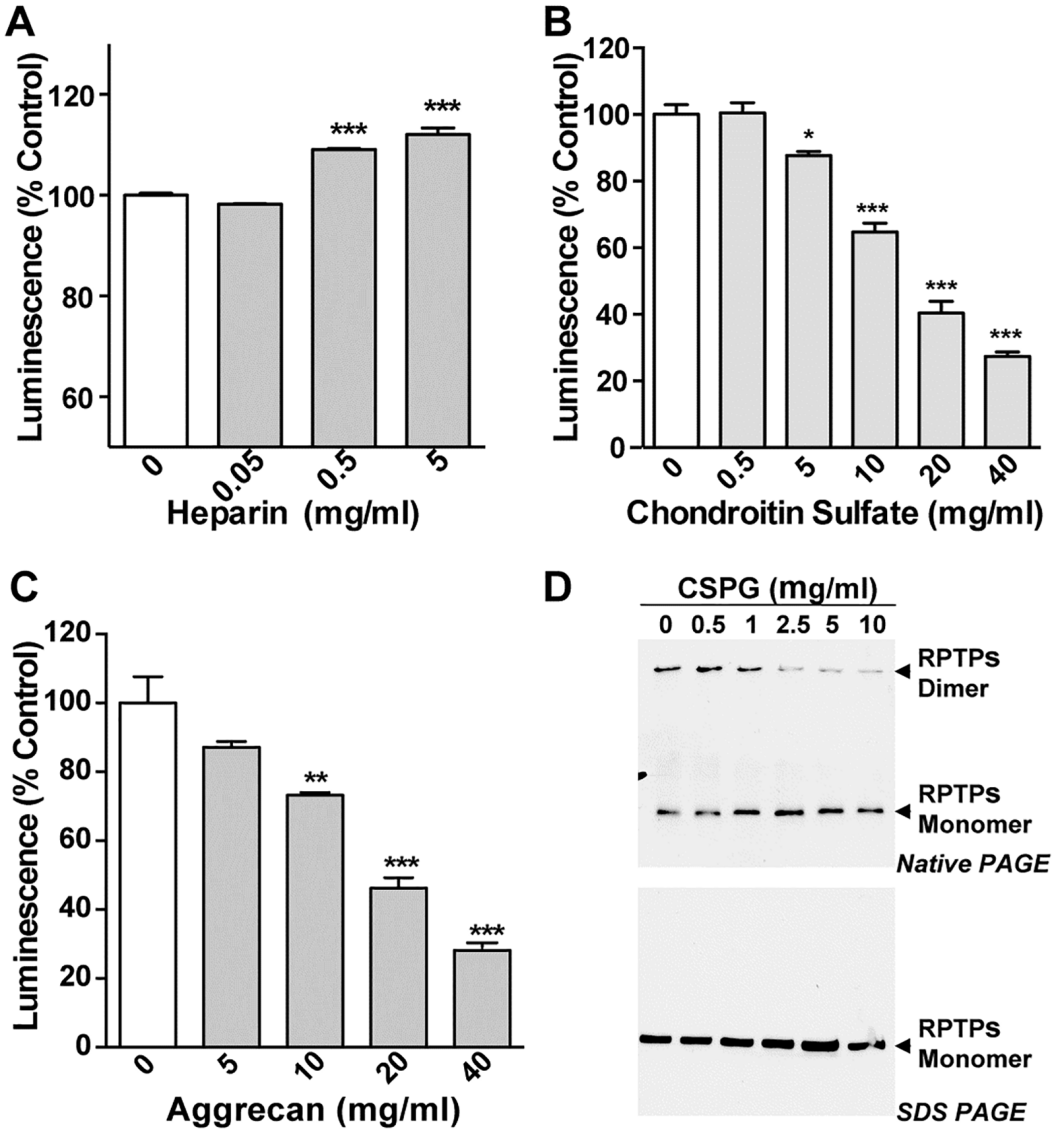
CSPG analogs prevent RPTPσ dimerization whereas Heparin induces it in living cells. (A-C) 293T/17 cells were co-transfected with RPTPσ N/C and 48 hr post-transfection, cells were treated with the indicated RPTPσ ligands for 5 min followed by luminescence measurement. Values are expressed as percentage vs control (without treatment) and the mean of three independent experiments performed in triplicates is given ± SD. * p < 0.05, ** p < 0.01, *** p < 0.001. (D) RPTPσ-transfected 293T/17 cells were treated with the indicated concentration of CSPG for 30 min and protein lysates were separated on a native (*top*) or SDS PAGE (*bottom*) followed by western blot analysis using the indicated antibodies.

Since dimerization and monomerization of RPTPσ could be seen in live cells, it opens the possibility that other ligands could modulate the dimerization status of RPTPσ through ligand/receptor interactions. Here we initially tested chondroitin sulfate (CS) and heparin, an analog of heparan sulfate since they are reported ligands of RPTPσ [4, 7, 16]. Moderately increased luciferase signals were seen in Heparin treated RPTPσ N/C expressing cells (Fig 2A). In contrast, addition of CS led to a dose-dependent decrease in luciferase activity (Fig 2B). Thus, these results suggested that these two known ligands interact differently with RPTPσ since CS seemingly acts on RPTPσ to prevent dimerization, whereas Heparin induces dimerization. We further examined the roles of CSPGs in the mediation of RPTPσ monomerization using Aggrecan, a member of the CSPG family protein. Adding this ligand to RPTPσ N/C expressing cells led to a decreased bioluminescence signal (Fig 2C), suggesting that Aggrecan inhibited RPTPσ dimerization in cells. In addition, a native gel showed that dimeric and monomeric forms of RPTPσ both exist in cells and when cells are treated with CSPG, it resulted in a switch from the dimer to the monomer form of RPTPσ (Fig 2D). Taken together our data demonstrates that CSPG and its analog promote RPTPσ monomerization.

**Fig 3:**
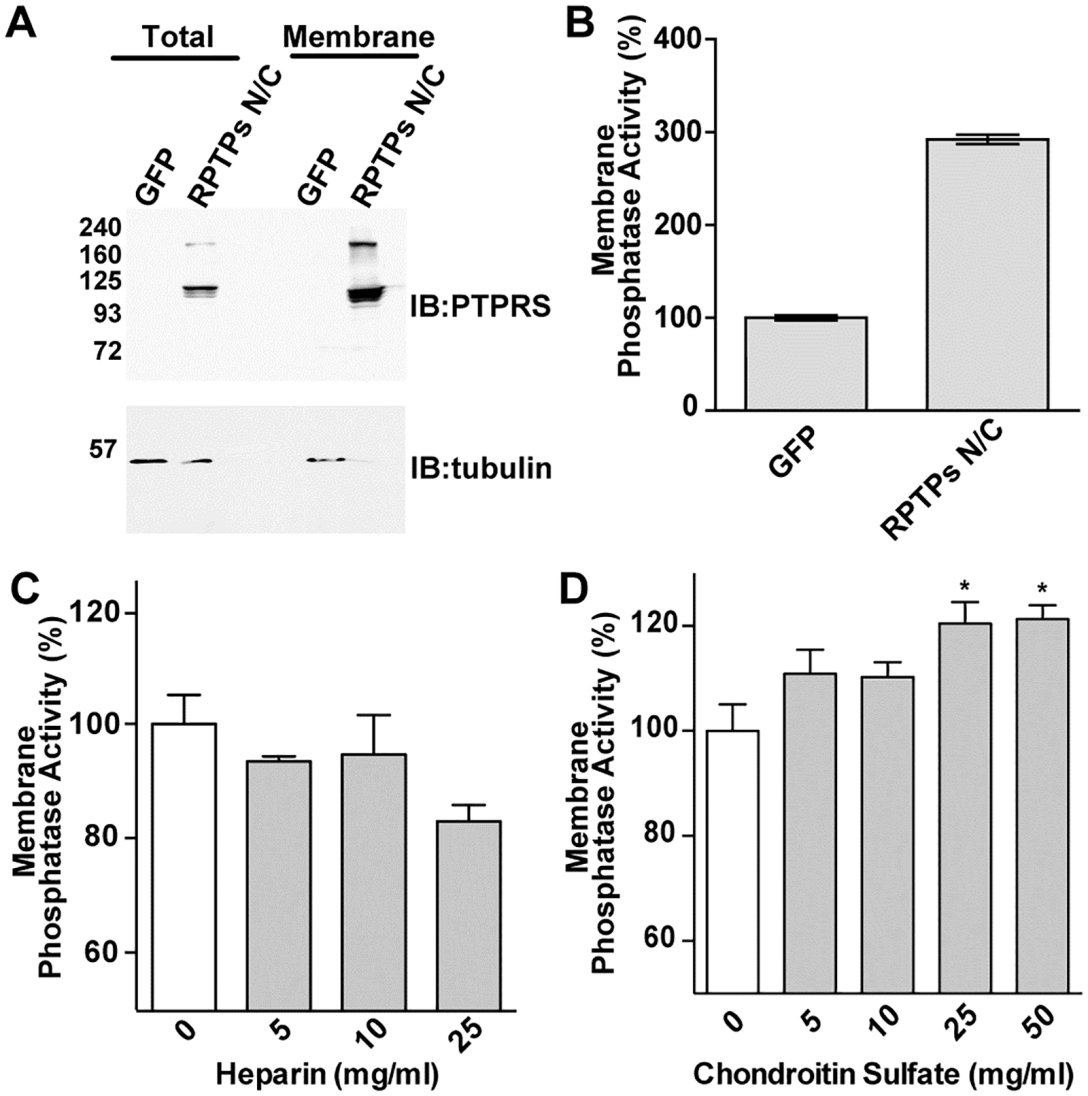
Chondrotin Sulfate increases RPTPσ activity. (A) 293T/17 cells were co-transfected with GFP control or RPTPσ N/C and 48 hrs post-transfection, cells were lysed and membrane fraction were isolated followed by western blot analysis. (B) The membrane fraction was incubated with the phosphatase substrate PNPP and release of PNP was measure using the Varioskan plate reader. Phosphatase activity values are expressed as percentage over GFP control. (C-D) 50 ug of isolated protein membranes fractions were incubated with different amount of Chondroitin Sulfate (CS) or heparin for 30 min at RT followed by the phosphatase activity assay. Values are expressed as percentage vs control (without treatment) and the mean of three independent experiments performed in triplicates is given ± SD. *, p <0.05.

Inactivation of the RPTPs CD45 and RPTPα has been show to be mediated by receptor dimerization [12–15]. However, it is unknown if this mechanism also applies to RPTPσ. Since both CS and heparin have opposite effect on RPTPσ dimerization, we employed an *in vitro* phosphatase assay to test if their binding caused a similar variation in RPTPσ enzymatic activities. To that end, we isolated membrane fractions from cells transfected with full-length RPTPσ proteins followed by a phosphatase assay using the artificial substrate pNPP. Immunoblotting showed that RPTPσ was enriched in the membrane fraction (Fig 3A). In addition, the membrane fraction from RPTPσ-transfected cells displayed a three-fold increase in phosphatase activity compared to cells transfected with a GFP expression vector (Fig 3B), indicating that full-length RPTPσ protein is a functional phosphatase in the membrane fraction. Membrane fractions derived from RPTPσ expressing cells were treated with either heparin or CS and further subjected to phosphatase assay as described above. Although we did not observe significant changes of phosphatase activity in presence of Heparin, CS treatment promoted phosphatase activity (Fig 3D). This strongly suggests that CS acts by inducing RPTPσ monomerization to increase its *in vivo* phosphatase activity.

### Antibody against the extracellular domain of RPTPσ promotes axonal growth

**Fig 4:**
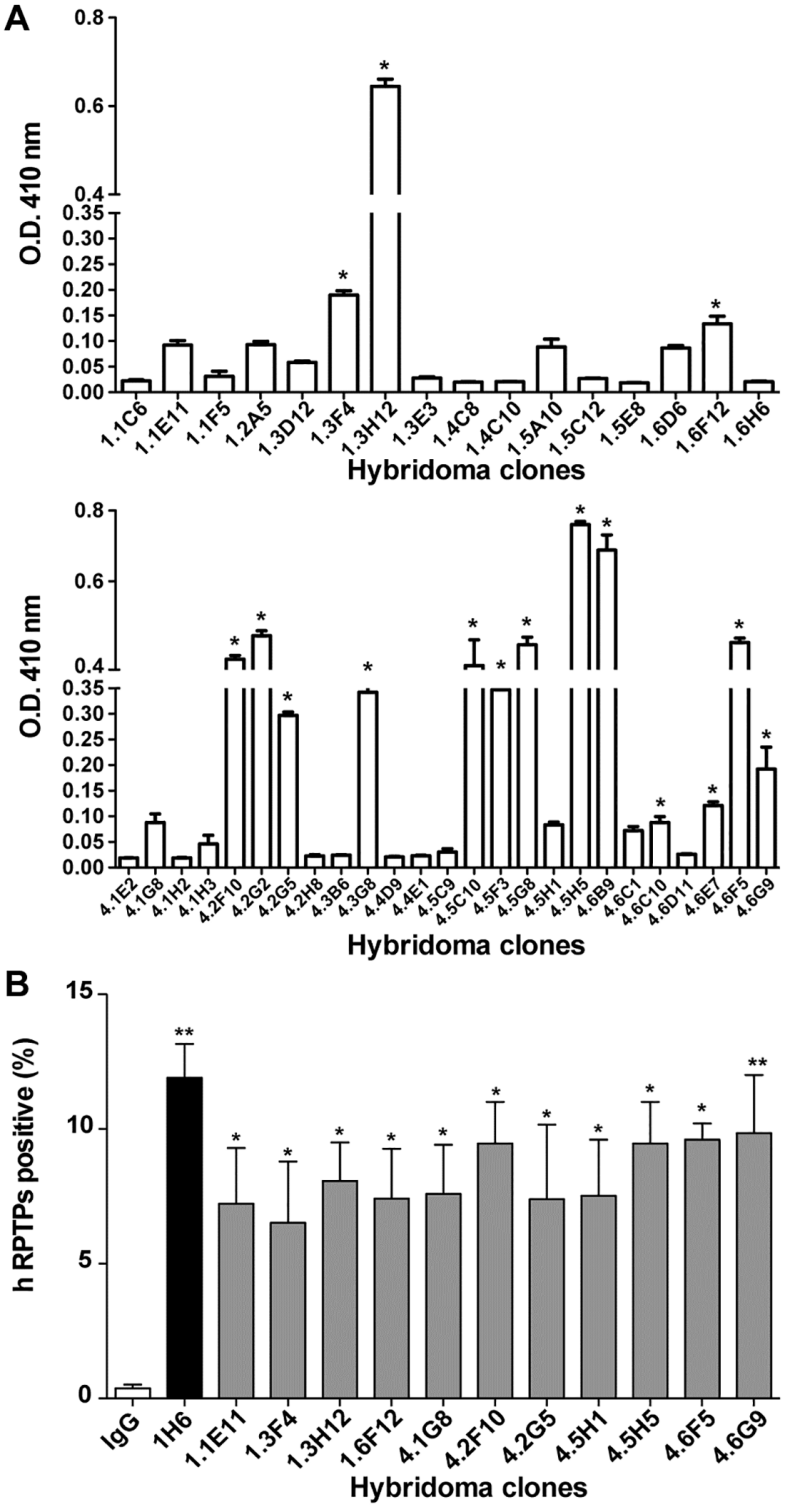
Generation of antibodies targeting the extracellular domain of human RPTPσ. (A) ELISA assay using peptides from human RPTPσ extracellular region coated in 96 well plates and incubated with the supernatant of hybridomas. (B) COS7 cells were transfected with human RPTPσ (hRPTPσ) and FACS analysis were performed using the supernatant of 11 positives hybridomas identified by ELISA. IgG is the negative control whereas the commercially available clone1H6 is the positive control. hRPTPσ positive represent the percentage of transfected cells detected expressing hRPTPσ on the cell surface. All experiments were performed in triplicates for a total of three experiments, which gave similar results. One representative experiment is given ± SD. * p < 0.05, ** p < 0.01.

In an effort to generate antibodies targeting the extracellular domain of RPTPσ, we generated and screened hybridoma clones to obtain 17 positive clones revealed by ELISA (Fig 4A). Furthermore, COS7 cells transiently overexpressing RPTPσ were analyzed by Fluorescent Activated Cell Sorting (FACS) to identify which of those antibodies produced by these hybridoma cells had the capacity to recognize the native structure of the extracellular domain of RPTPσ and we identified eleven clones that were positive by FACS analysis (Fig 4B).

**Fig 5:**
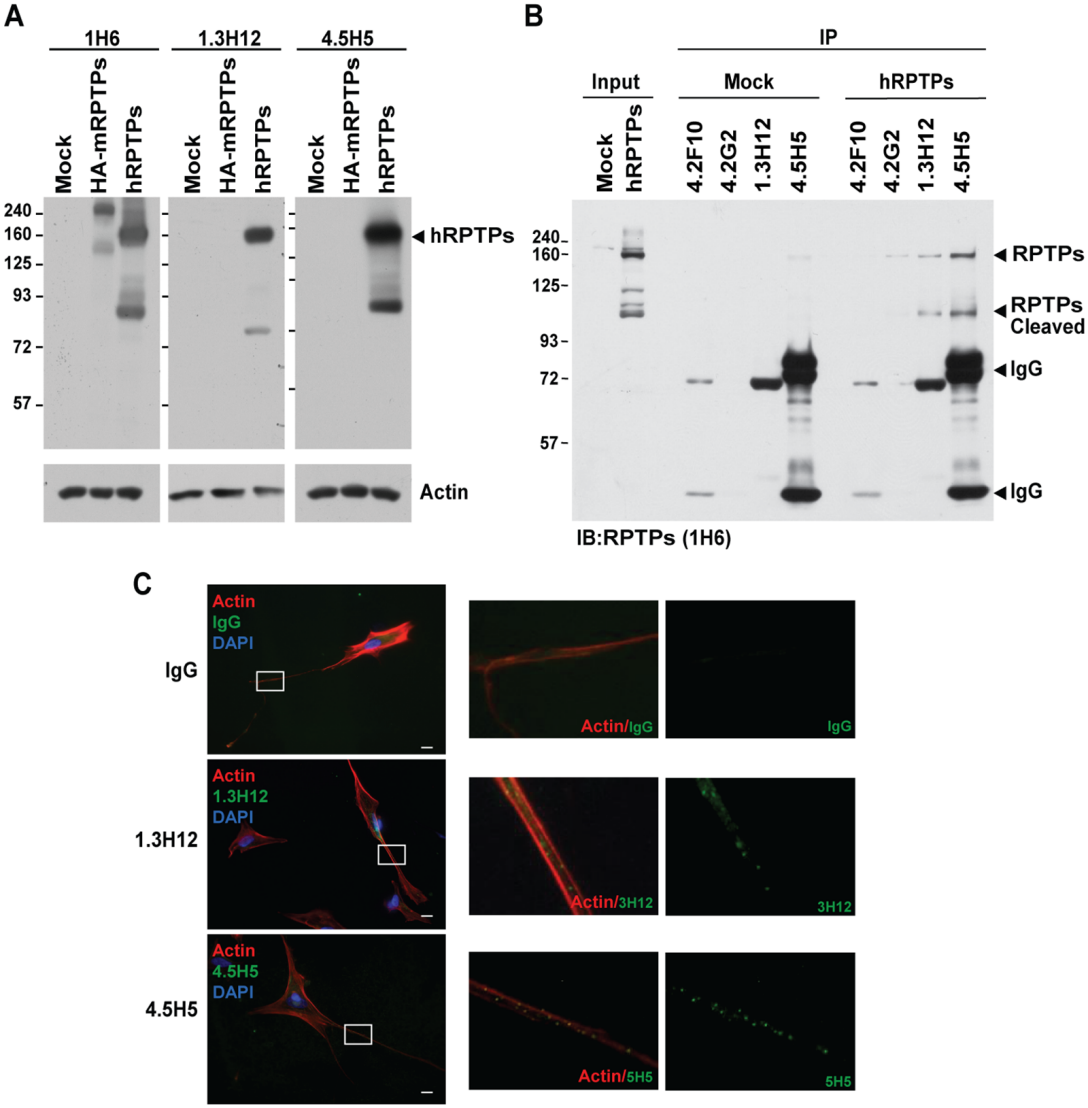
Characterization of purified monoclonal antibodies targeting RPTPσ. COS7 cells were transfected with either human RPTPσ (hRPTPσ) or mouse RPTPσ (HA-mRPTPs) and 24 hrs post-transfection, western blot (A) and immunoprecipitation (B) analysis was performed using the indicated purified monoclonal antibodies. Antibody 1H6 reacted with both forms was used as positive control. Actin was the loading controls. **C**: Differentiated SH-SY5Y cells were immunostained using the purified 1.3H12 or 4.5H5 monoclonal antibodies for RPTPσ (green), Dapi for the nucleus (blue) and Phalloidin for actin (red). IgG was used as negative control.

Based on ELISA and FACS analysis, 1.3H12 and 4.5H5 antibodies were selected because they displayed the highest sensitivity for RPTPσ. They were purified for further characterization. The two antibodies reacted only with the human form of RPTPσ but not the mouse isoform (Fig 5A). Furthermore, we confirmed that they recognize native protein in immunoprecipitation experiments with strong specificity since both 1.3H12 and 4.5H5 only recognize human RPTPσ but not mouse one (Fig 5B). In addition, immunofluorescence analysis detected the endogenous hRPTPσ expression in SH-SY5Y human glioblastoma cells that when differentiated display neuronal morphology and extensive axonal outgrowth. Since RPTPσ is a cell-surface protein, these antibodies are able to recognize the extracellular fragment of RPTPσ in non-permeabilized conditions. RPTPσ exhibited a punctate staining pattern on the axon-like structures of differentiated SH-SY5Y cells stained with 1.3H12 and 4.5H5 antibodies but not IgG control (Fig 5C). This staining pattern is consistent with that of mouse RPTPσ reported in a previous study [9], further confirming that RPTPσ localizes preferentially on the surface of axons.

**Fig 6:**
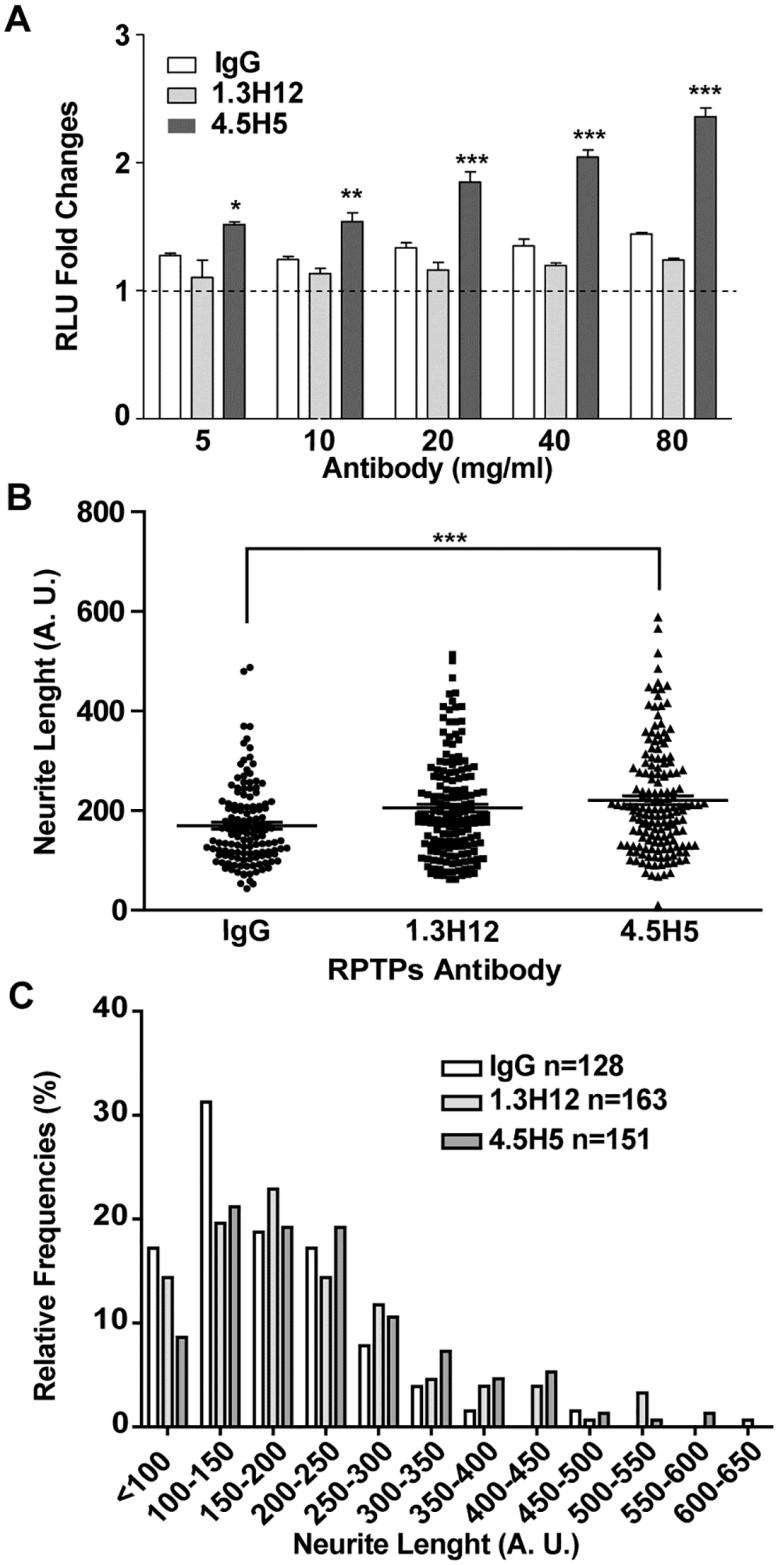
RPTPσ 4.5H5 monoclonal antibody induces RPTPσ dimerization and increases neurite outgrowth in SH-SY5Y cells. (A) 297T/17 cells were transfected with hRPTPσ N/C and 48 hrs post-transfection, cells were treated with various amount of the indicated RPTPσ monoclonal antibody followed by luminescence measurement. IgG was used as negative control. Values are expressed as fold changes vs respective IgG control. All experiments were performed in triplicates for a total of three experiments, which gave similar results. One representative experiment is given ± SD. * p < 0.05, ** p < 0.01, *** p < 0.001 (B) SH-SY5Y neuroblastoma cells were treated with human RPTPσ antibodies (10μg/ml) in the differentiation medium. 96 hr post-treatment cells were subjected to image analysis using the NeuronJ program and neurite length and the distribution of neurite length (C) were determined. A.U.: arbitrary unit. One representative experiment of a total of three experiments is shown. ***, p < 0.001.

We tested whether antibodies modulate the dimerization of RPTPσ using the split luciferase assay. Our result shows that the 4.5H5 antibody but not the 1.3H12 antibody significantly increased luminescence, indicating that 4.5H5 was able to significantly induce the dimerization of RPTPσ (Fig 6A). Finally, to determine if these antibodies have the capacity to regulate neurite growth, differentiated SH-SY5Y cells were incubated in the presence of 1.3H12, 4.5H5 and IgG control respectively for 96 hrs. The result shows that SH-SY5Y cells treated with the 4.5H5 antibody bore longer axons than cells treated with the IgG control or 1.3H12 antibody (Fig 6B and 6C). Overall, these data suggest that the 4.5H5 antibody could potentially be use for therapeutic treatment of nerve injury and possibly other neurodegenerative diseases.

## Discussion

RPTPσ is one of the three members of the type II sub-family of receptor PTPs [24]. Our group and others have shown over the past decades that RPTPσ is a potential target for the development of novel therapies for spinal cord injury and multiple neurodegenerative diseases [3, 4, 6, 7]. Moreover, we recently reported that RPTPσ inhibition could improve memory [5]. These exciting findings underlie the need for a screening assay that can monitor inhibition of the catalytic activity mediated by RPTP dimerization.

We selected the split luciferase assay, which can be used to monitor protein-protein interactions in intact cells [18, 20–22]. We have demonstrated that this assay can be used to screen for ligands, potentially for small molecule inhibitors, and for anti-RPTPσ antibodies that may regulate RPTPσ dimerization. Surprisingly, validation of this assay also revealed some insight into the structural characteristics of RPTPσ in cells. In live cells, RPTPσ exists predominantly as a dimer. Our results showed that the dimerization of RPTPσ is not solely attributed to the intracellular wedge domain, as previous studies have suggested [13, 15, 25], because the membrane anchored extracellular domain of RPTPσ is still capable of forming a dimer, withtout the intracellular domain. The DIDII phosphatase domain of RPTPσ without the wedge domain displayed fairly low but noticeable luminescence suggesting that the DIDII domain mainly presents as a monomer. On the other hand, different versions of RPTPσ pairings assayed by the luciferase system suggest that the dimerization of RPTPσ is mainly attributed to the extracellular domain and not to the intracellular domain. It also suggests that the dimerization of RPTPσ is not only regulated by the wedge domain and the DII domain [13, 17], but also by ligands/receptor interactions.

CSPGs and HSPGs are newly identified ligands of RPTPσ [4, 6, 7] and crystallographic analysis suggests that CSPGs prevent RPTPσ dimerization, whereas HSPGs induce RPTPσ clustering [26]. In addition, both CSPGs and HSPGs share the same binding site on the first Ig-like domain of RPTPσ suggesting that they compete for RPTPσ binding, inducing opposite effects on axonal outgrowth [26]. CSPGs inhibit axon outgrowth in RPTPσ WT neurons. This effect was significantly diminished in RPTPσ KO neurons consistent with previous reports [3, 4, 6, 27]. In addition, HSPGs dramatically promoted axon outgrowth in WT but not KO neurons, suggesting that this promoting effect by HSPGs depends on RPTPσ. Both CSPGs and HSPGs have a similar affinity for RPTPσ; therefore, the CSPGs/HSPGs ratio is the key factor in determining RPTPσ dimerization status.

Using our screening system, we confirmed that CSPG is a ligand of RPTPσ and that CSPGs prevent RPTPσ from dimerizing, resulting in its activation. However, HSPG-mediated RPTPσ dimerization seems moderate in our system. One possible explanation is that RPTPσ exists primarily as a dimer in cells and it is more difficult to induce further dimer formation and inhibition of phosphatase activity.

Our results indicate that the ligand-dependent switch between the monomeric and the dimeric forms of RPTPσ provides an important means of regulating RPTPσ function. There is still much to explore regarding the interaction of CSPGs and HSPGs with RPTPσ from a structural point of view. The crystal structure of the extracellular domain of RPTPσ has been resolved and gives us some structural insights into ligands/receptor interactions [26]. There is still an urgent need to acquire the co-crystal structure of CSPGs/RPTPσ and that of HSPG/RPTPσ to elucidate how CSPGs and HSPGs affect the dimerization status of RPTPσ. Such findings could be applied to pharmaceutical development of small molecule inhibitors or therapeutic antibodies to inhibit RPTPσ activity. In a therapeutic context, inhibitors blocking the interaction between CSPGs and RPTPσ may not be sufficient; a putative RPTPσ-targeted drug may require the capability to induce dimerization of RPTPσ in order to completely inhibit RPTPσ activity. Thus, a monoclonal antibody-based approach, as we proposed here, could be ideal.

The development of small molecule inhibitors has been extremely difficult for researchers studying protein tyrosine phosphatases. Traditional PTP inhibitors mostly target the catalytic domain of PTPs, which is highly conserved among all PTPs; hence they generally suffer from lack of specificity toward its targeted PTPs. Recently, Silver’s group has developed a wedge domain peptide-based RPTPσ inhibitor and showed its effectiveness in a spinal cord injury animal model [28]. Still, the specificity of this inhibitor could be an issue since most receptor tyrosine phosphatases possess a well-conserved wedge domain. An alternative approach is to use specific allosteric inhibitors [29] or, as shown here, antibodies targeted to the extracellular domain of RPTPs.

Antibodies of the IgG class generally mediate receptor dimerization since they are bivalent in nature, facilitating the non-covalent crosslinking of two receptors. However, cumulative evidence indicates that the structures of the receptor ectodomains have to be considered when designing therapeutic antibodies. Indeed, taking lessons from therapeutic antibodies directed against EGFR [30], we recognize that it is similarly challenging to design therapeutic antibodies that not only induce dimerization of RPTPσ, but also simultaneously inactivate it. The 4.5H5 antibody gives us some confidence that this type of high specificity antibody could be found for pharmaceutical applications. Finally, the RPTPσ split luciferase assay would provide a valuable system to screen these types of modulators in a high throughput format.

Collectively, data collected using our RPTPσ split luciferase system not only provide a cell-based platform to examine the genuine properties of RPTPσ in live cells, but also reveals its potential for the screening of ligands and potential therapeutic antibodies. This system could ultimately lead to discovery of novel RPTPσ inhibitors designed for the treatment of patients suffering from spinal cord injury or a broad array of neurodegenerative disorders.
